# Supplementary material for: Thermodynamic framework for assessing dissolutive wetting behaviors in metallic systems
Source: Nat Commun. 2025 Dec 3;17:313. doi: 10.1038/s41467-025-67008-9 (PMC12789441; doi:10.1038/s41467-025-67008-9)
Supplement: Supplementary file 1 — Supplementary Information [file 41467_2025_67008_MOESM1_ESM.pdf]

Supplementary Information file for

Thermodynamic framework for assessing dissolutive wetting behaviors in metallic systems

Youqing Sun[1,2,3], Shoufeng Yang[1,2*], Xinghong Cai[3,4*], Zhongfu Cheng[3*], Wantong Chen[3], Nele Moelans[3], Muxing Guo[3], David Seveno[3]

Affiliations

1 Chongqing Institute of Green and Intelligent Technology, Chinese Academy of Sciences, Chongqing 400714, China

2 University of Chinese Academy of Sciences, Chongqing School, Chongqing 400714, PR China

3 Department of Materials Engineering, KU Leuven, Kasteelpark Arenberg 44, 3001 Leuven, Belgium

4 Chongqing Key Laboratory for Advanced Materials and Technologies of Clean Energies, School of Materials and Energy, Southwest University, Chongqing 400715, China

E-mail: shoufeng.yang@cigit.ac.cn; caixinghong@cqust.edu.cn; zhongfu.cheng@kuleuven.be

Table of contents

Supplementary Fig. 1 Unedited versions of the images for Fig. 1 in the main text.

Supplementary Fig. 2 Repeated experiments for Cu/Ni, Cu/Ti and Cu/Fe systems.

Supplementary Fig. 3 Unedited versions of the images for Fig. 2 and 3 in the main text.

Supplementary Fig. 4 The relationship between fraction of solid solutions and C_R with different Fe compositions at the L/S interface for Cu/Fe.

Supplementary Fig. 5 Schematical illustration of the numerical approach showing the formation of a double solute atom system starting from the Cu network.

Supplementary Fig. 6 Schematical illustration of the numerical approach showing the formation of a defect system starting from the Cu network.

Supplementary Fig. 7 The densities of states of the diatomic systems.

Supplementary Fig. 8 The densities of states of the defect systems.

Supplementary Table 1 Calculated heat of solution (H_S, eV), structural (H_{SC}, eV) and chemical (H_{CC}, eV) contributions of Ti, Ni, and Fe in Cu crystal structures (diatomic system).

Supplementary Table 2 Calculated heat of solution (H_S, eV), structural (H_{SC}, eV) and chemical (H_{CC}, eV) contributions of Ti, Ni, and Fe in the defective Cu crystal structures.

Supplementary Fig. 9 Observed steps and fitting errors during spreading.

Supplementary Fig. 10 Step flow behaviors and quenched microstructures of the Au/Pt system.

Supplementary Fig 11 Step flow behaviors and quenched microstructures of the Ag/Cu system.

Supplementary Fig. 12 Step-flow behaviors and quenched microstructures of the Sn/Cu system.

Supplementary Fig. 13 Spreading behavior and quenched microstructures of the Ag/Ni system.

Supplementary Fig. 14 Spreading behavior and quenched microstructures of the Sn/Fe system.

Supplementary Fig. 15 Spreading behavior and quenched microstructures of the Ag/Ti system.

Supplementary Fig. 16 Illustration of the non-contact heating experiments.

Supplementary Fig. 17 Size effects of droplets in non-contact heating experiments.

Supplementary Fig. 18 Illustration of the spreading process.

Unedited versions of the images for Fig. 1

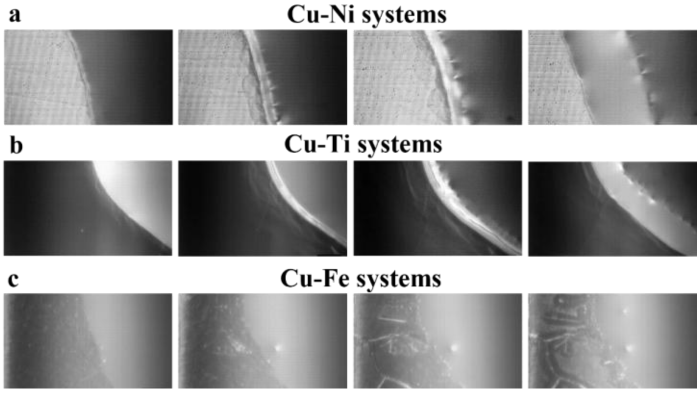

Supplementary Fig. 1 | Unedited versions of the images for Fig. 1 in the main text. a Cu/Ni, (**b**) Cu/Ti. and (**c**) Cu/Fe systems.

Repeated experiments

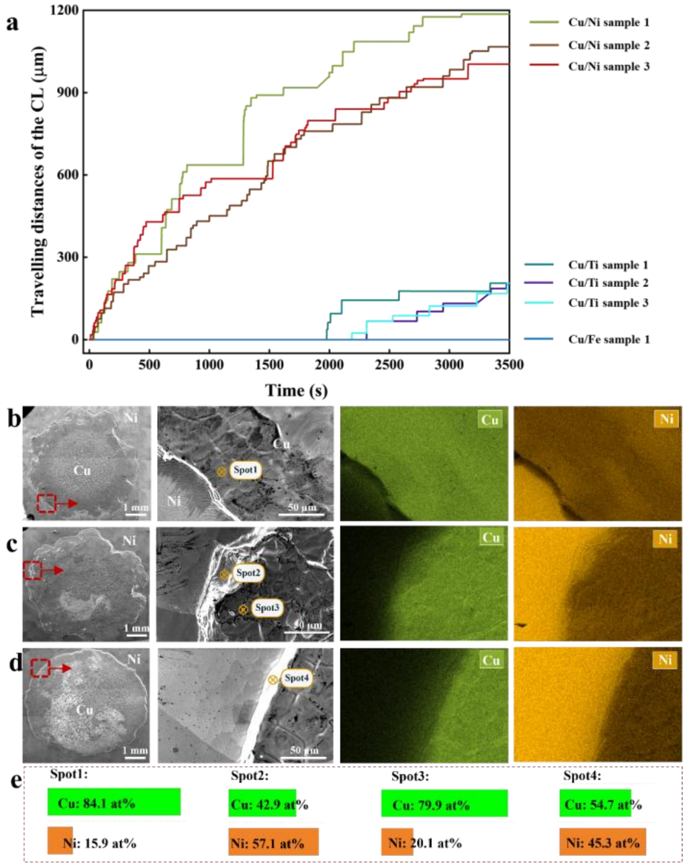

Supplementary Fig. 2 | Repeated experiments for Cu/Ni, Cu/Ti and Cu/Fe systems. a Travelling distances of the contact line (CL) during the isothermal stage (contact heating methods) for the three systems. **b, c, d** Secondary electron (SE) pictures and (**e**) the Energy Dispersive Spectroscopy (EDS) point analysis for quenched Cu/Ni systems. The red dashed rectangle in (**b, c, d**) marks the region shown at higher magnification. Source data for (**a**) are provided as a Source data file.

Unedited versions of the images for Fig. 2 and 3

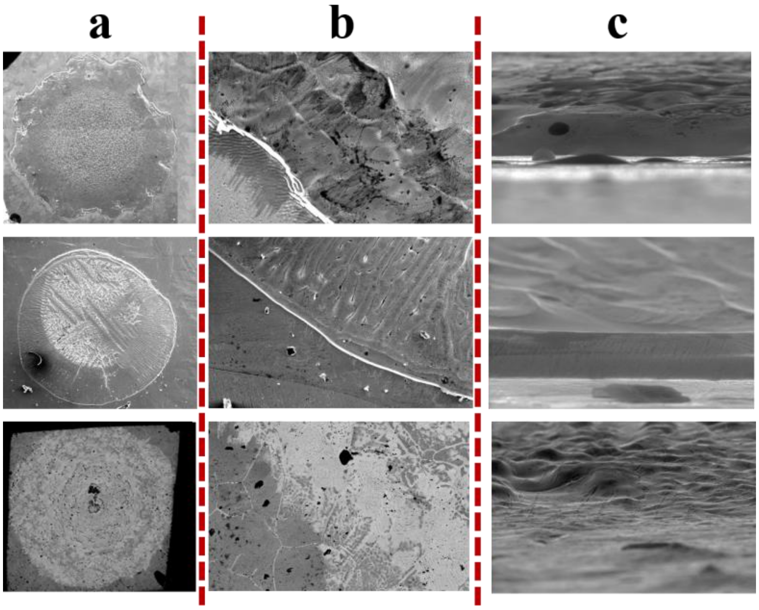

Supplementary Fig. 3 | Unedited versions of the images for Fig. 2 and 3 in the main text. a Top-view secondary electron (SE) images. **b** enlarged SE images near the contact line (CL). **c** side-view SE images.

Theoretical C_R threshold preventing the step flow mechanism

Assuming equilibrium solidification at the liquid Cu/Solid Fe interface, the fraction of solid solutions (f_S) in C_R can be estimated using the lever rule (Equation S3) :

$$f_S = \frac{C_0 - C_L}{C_R} = \frac{C_0 - 0.047}{C_R} \quad \text{(S3)}$$

where C_0 is the Fe composition at the L/S interface. Based on quenched samples, $C_0 \approx 0.05$. As shown in Figure A15, complete solidification ($f_S \approx 1$) requires $C_R \approx 0.02$, and even for $C_0 = 0.15$, full solidification still requires $C_R \approx 0.10$. In contrast, the actual value C_R = 0.839 is far above this threshold, meaning that the CL cannot pin and the step-flow mechanism cannot occur.

We note, however, that this analysis is based on ideal equilibrium conditions. In reality, isothermal solidification may deviate from equilibrium[1,2], and partial solidification (e.g., $f_S \approx 0.8$) could still introduce “pinning” effects. Even so, the large C_R value of 0.839 clearly precludes activation of the step-flow mechanism.

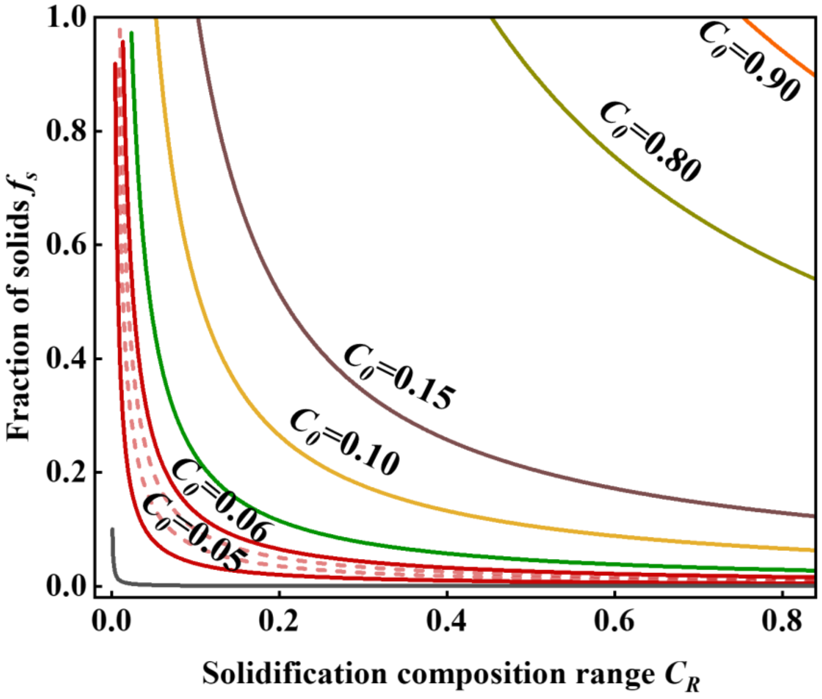

Supplementary Fig. 4 | The relationship between fraction of solid solutions and C_R with different Fe compositions at the L/S interface for Cu/Fe. C_0 is the Fe composition at the L/S interface. C_R is solidification composition range. Source data are provided as a Source data file.

Effects of multi-atoms and defects on atomic interactions

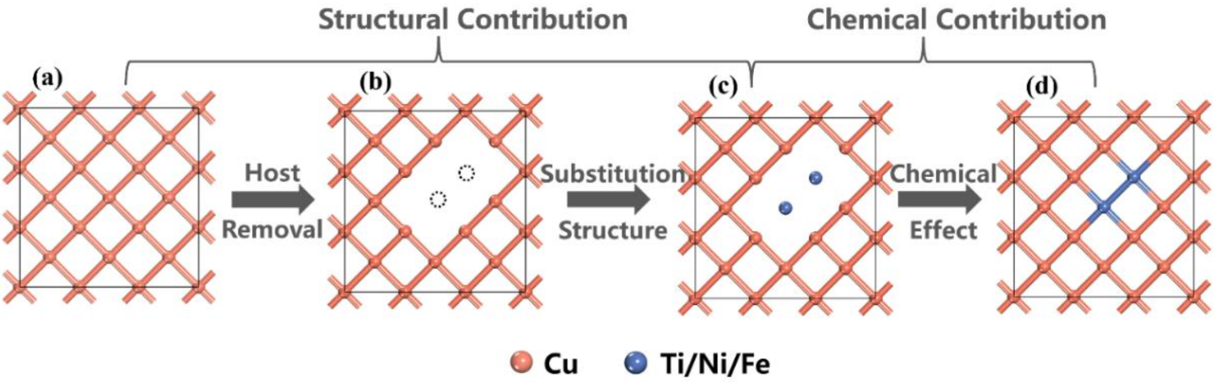

Supplementary Fig. 5 | Schematical illustration of the numerical approach showing the formation of a double solute atom system starting from the Cu network. From (**a**) to (**b**), formation of two atom vacancies by removing two Cu atoms. From (**b**) to (**c**), insertion of two solute atoms. From (**c**) to (**d**), formation of chemical bonds between the solvent and solute atoms. The atomic coordinates are provided as Supplementary Data 1.

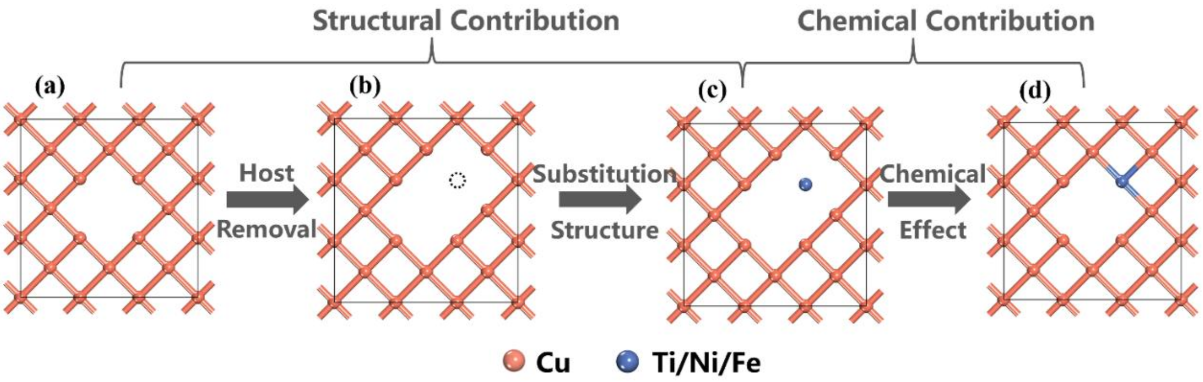

Supplementary Fig. 6 | Schematical illustration of the numerical approach showing the formation of defect systems starting from the Cu network. From (**a**) to (**b**), formation of an atom vacancy defect by removing two Cu atoms. From (**b**) to (**c**), insertion of a solute atom. From (**c**) to (**d**), formation of chemical bonds between the solvent and the solute atom. The atomic coordinates are provided as Supplementary Data 1.

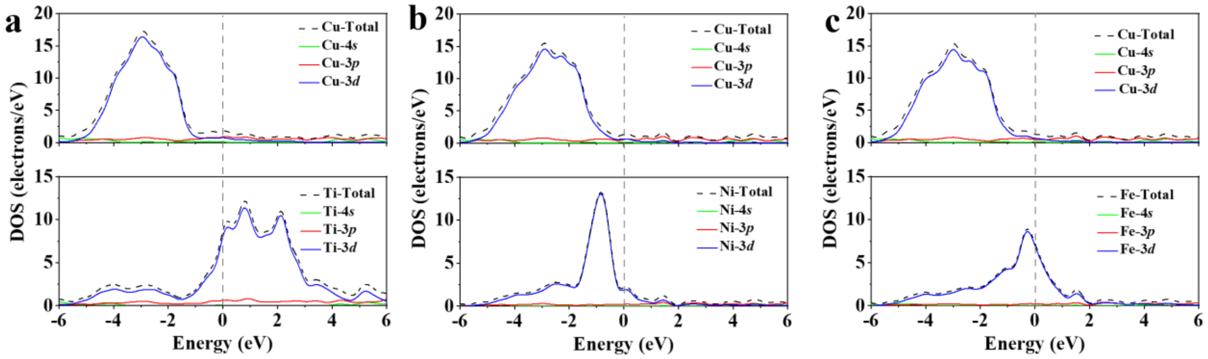

Supplementary Fig. 7 | The densities of states of the diatomic systems. a Cu/Ti. **b** Cu/Ni. **c** Cu/Fe. Source data for (**a-c**) are provided as a Source data file.

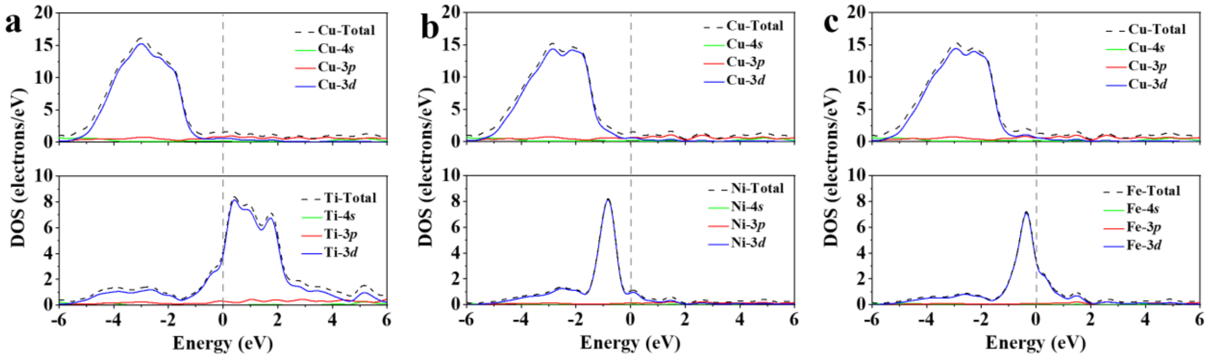

Supplementary Fig. 8 | The densities of states of the defect systems. a Cu/Ti. **b** Cu/Ni. **c** Cu/Fe. Source data for (**a-c**) are provided as a Source data file.

Supplementary Table 1. Calculated heat of solution (H_S, eV), structural (H_{SC}, eV) and chemical (H_{CC}, eV) contributions of Ti, Ni, and Fe in Cu crystal structures (diatomic system).

Solutes	H_S (eV)	H_{SC} (eV)		H_{CC} (eV)
		E_{HR}	E_{SS}	
Ti	-0.05	2.08	0.50	-2.63
Ni	0.22	2.08	0.06	-1.92
Fe	0.56	2.08	0.10	-1.62

Supplementary Table 2. Calculated heat of solution (H_S, eV), structural (H_{SC}, eV) and chemical (H_{CC}, eV) contributions of Ti, Ni, and Fe in the defective Cu crystal structures.

Solutes	H_S (eV)	H_{SC} (eV)		H_{CC} (eV)
		E_{HR}	E_{SS}	
Ti	0.08	0.90	0.24	-1.06
Ni	0.35	0.90	0.02	-0.57
Fe	0.73	0.90	0.02	-0.20

Fitting errors and observed 'steps' during spreading

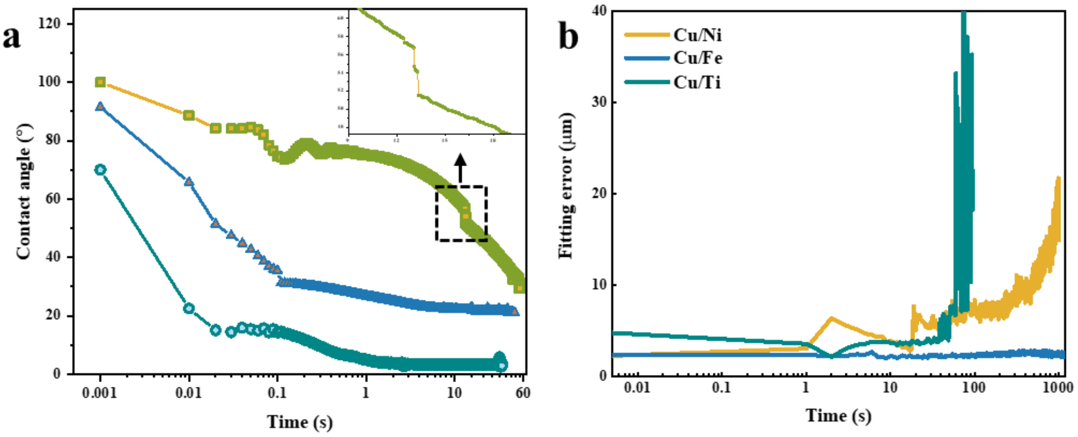

Supplementary Fig. 9 | Observed steps and fitting errors during spreading. a The sudden 'step' of spreading as a result of step flow mechanism is observed. **b** Fitting errors indicates an inaccurate fitting from 100 and 1000 s in Cu/Ti and Cu/Ni systems, respectively. Source data for (**b**) are provided as a Source data file.

Spreading, microstructure and thermodynamics in other metallic systems

Au on Pt at 1150 °C:

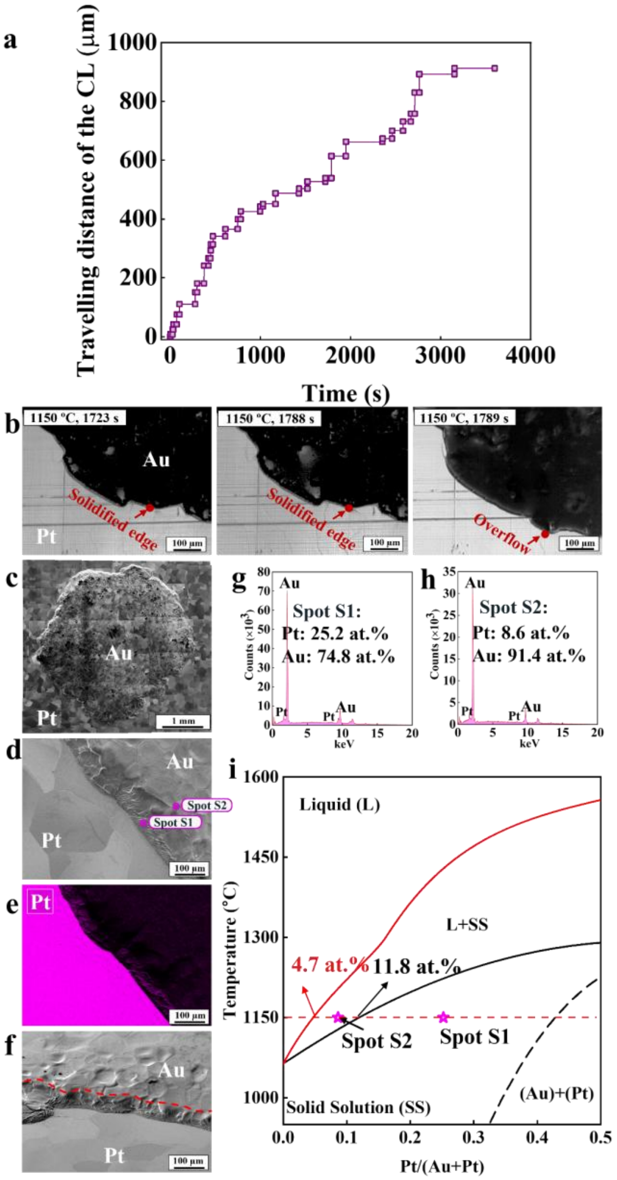

Supplementary Fig. 10 | Step flow behaviors and quenched microstructures of the Au/Pt system. a Traveling distances of the contact line (CL) indicates a rapid initiation of the step flow mechanism, as validated by (**b**) in-situ observations. Top-view secondary electron (SE) images of the quenched sample reveal (**c**) a flattened Au droplet and (**d**) a sharp edge at the CL. Energy-dispersive spectroscopy (EDS) mapping shows **e** the distribution of Pt within the Au droplet. **f** A side-view SE image further confirms the sharp edge, with a red dashed line representing its upper boundary. **g, h** Pt compositions measured near the CL and at the sharp edge, as marked in (**d**). The corresponding (**i**) phase diagram suggests that the solid solution can rapidly form due to small solubility of substrate elements and solidification composition range. Source data for (**a**) and (**g, h**) are provided as a Source data file.

Ag on Cu at 1000 °C:

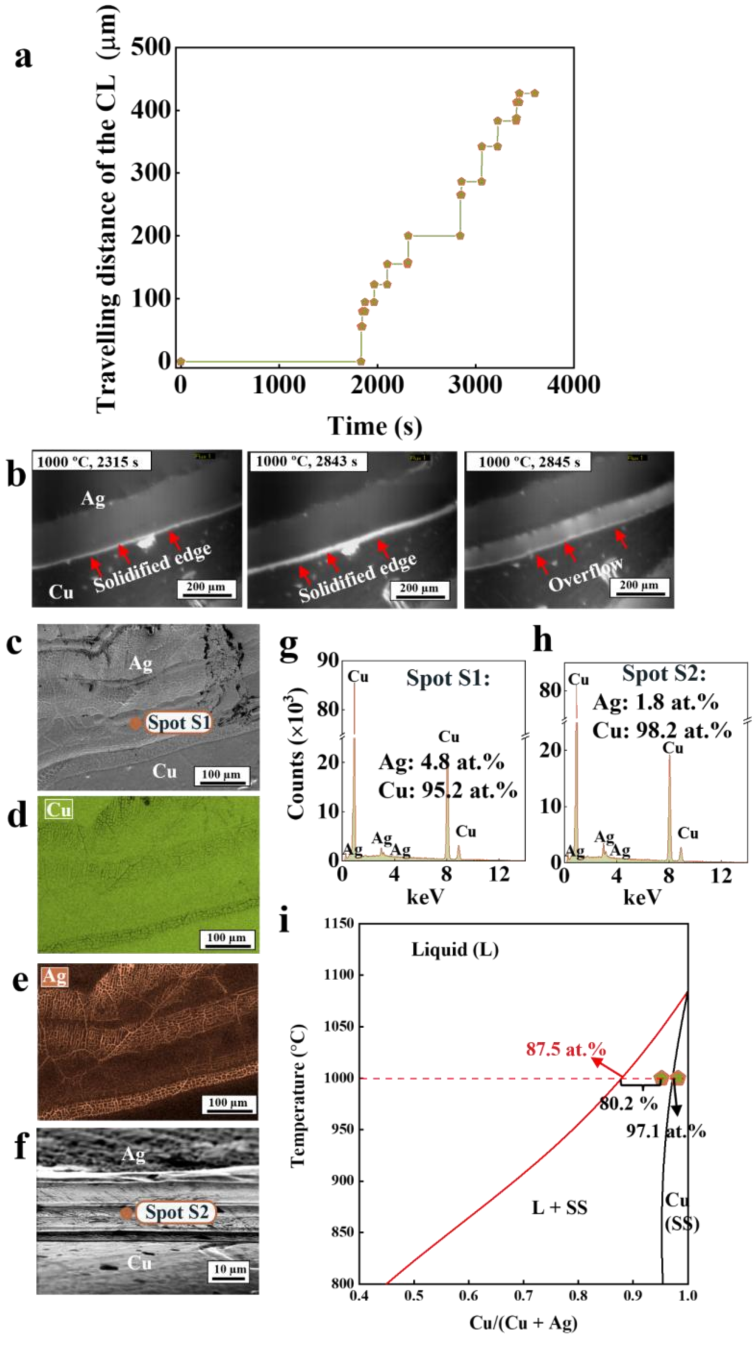

Supplementary Fig. 11 | Step flow behaviors and quenched microstructures of the Ag/Cu system. a Travelling distances of the contact line (CL) indicates a late presence of the step flow mechanism (after 1500 s), as validated by (**b**) in-situ observations. Top-view secondary electron (SE) images of the quenched sample reveal (**c**) a sharp edge at the CL. Energy-dispersive spectroscopy (EDS) mapping shows the distribution of (**d**) Cu within (**e**) the Ag droplet. **f** A side-view SE image further confirms the sharp edge. **g, h** Cu compositions measured near the CL and at the sharp edge, as marked in (**c, f**). The corresponding (**i**) phase diagram suggests that the solid solution can form after reaching solubility of substrate elements due to small solidification composition range. Source data for (**a**) and (**g, h**) are provided as a Source data file.

Sn on Cu at 800 °C:

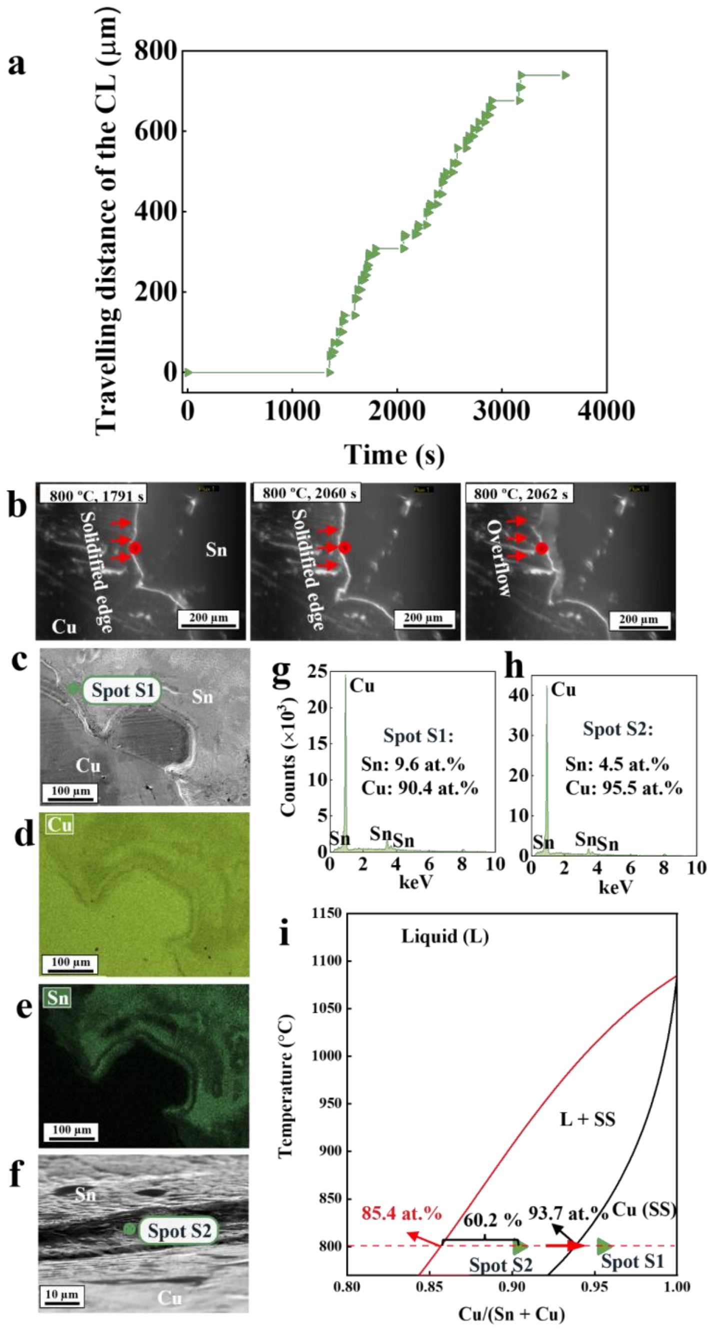

Supplementary Fig. 12 | Step-flow behaviors and quenched microstructures of the Sn/Cu system. a Travelling distances of the contact line (CL) indicates a late presence of the step flow mechanism (after 1000 s), as validated by (**b**) in situ observations. Top-view secondary electron (SE) images of the quenched sample reveal (**c**) a sharp edge at the CL. Energy-dispersive spectroscopy (EDS) mapping shows the distribution of (**d**) Cu within (**e**) the Sn droplet. **f** A side-view SE image further confirms the sharp edge. **g, h** Cu compositions measured near the CL and at the sharp edge, as marked in (**c, f**). The corresponding (**i**) phase diagram suggests that the solid solution can form after reaching solubility of substrate elements due to small solidification composition range. Source data for (**a**) and (**g, h**) are provided as a Source data file.

Ag on Ni at 1150 °C:

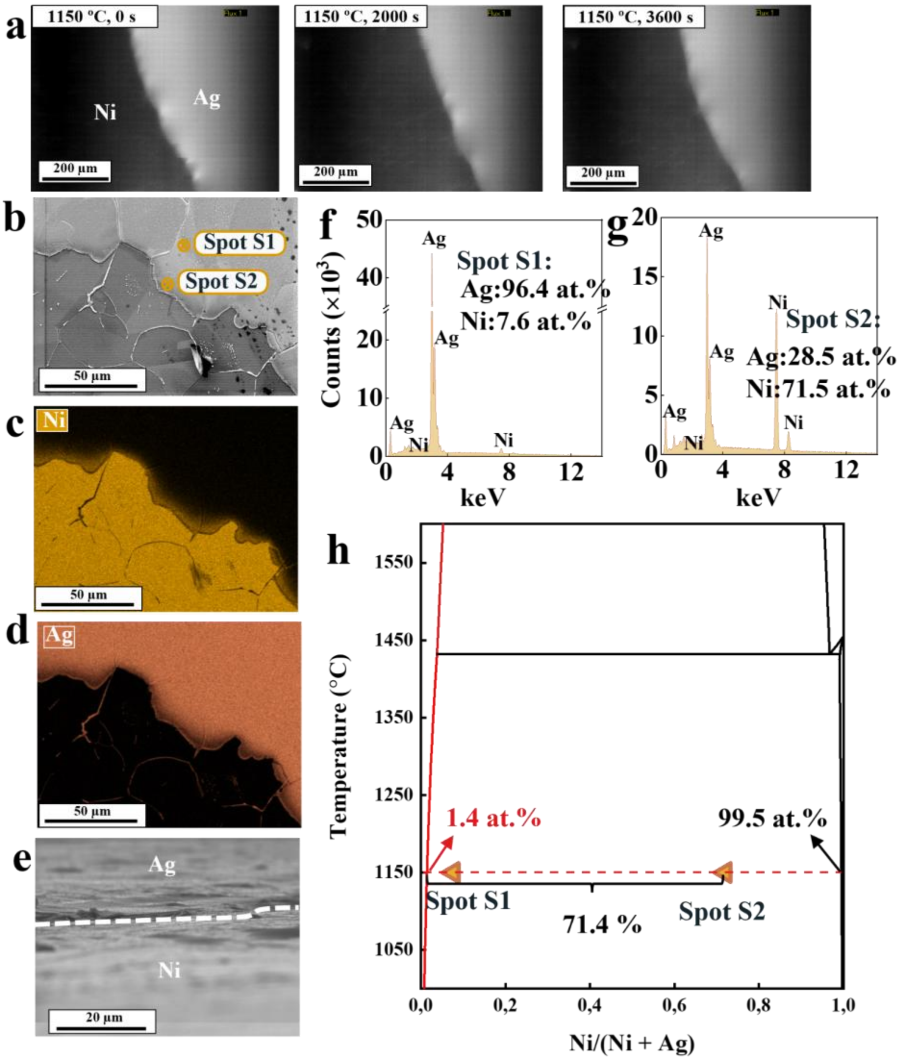

Supplementary Fig. 13 | Spreading behavior and quenched microstructures of the Ag/Ni system. a In-situ observations indicates that the contact line (CL) remains stationary during the isothermal stage. **b** Top-view secondary electron (SE) images of the quenched sample reveal the absence of sharp edges at the CL. Energy-dispersive spectroscopy (EDS) mapping shows the minor distribution of (**c**) Ni within (**d**) the Ag droplet. **e** A side-view SE image further confirms the absence of a sharp edge at the CL (white dash line). **f, g** Ni compositions measured near the CL, as marked in (**b**). The corresponding (**h**) phase diagram suggests that the formation of solid solutions at the liquid/solid interface is impeded due to the large solidification composition range. Source data for (**f, g**) are provided as a Source data file.

Sn on Fe at 950 °C:

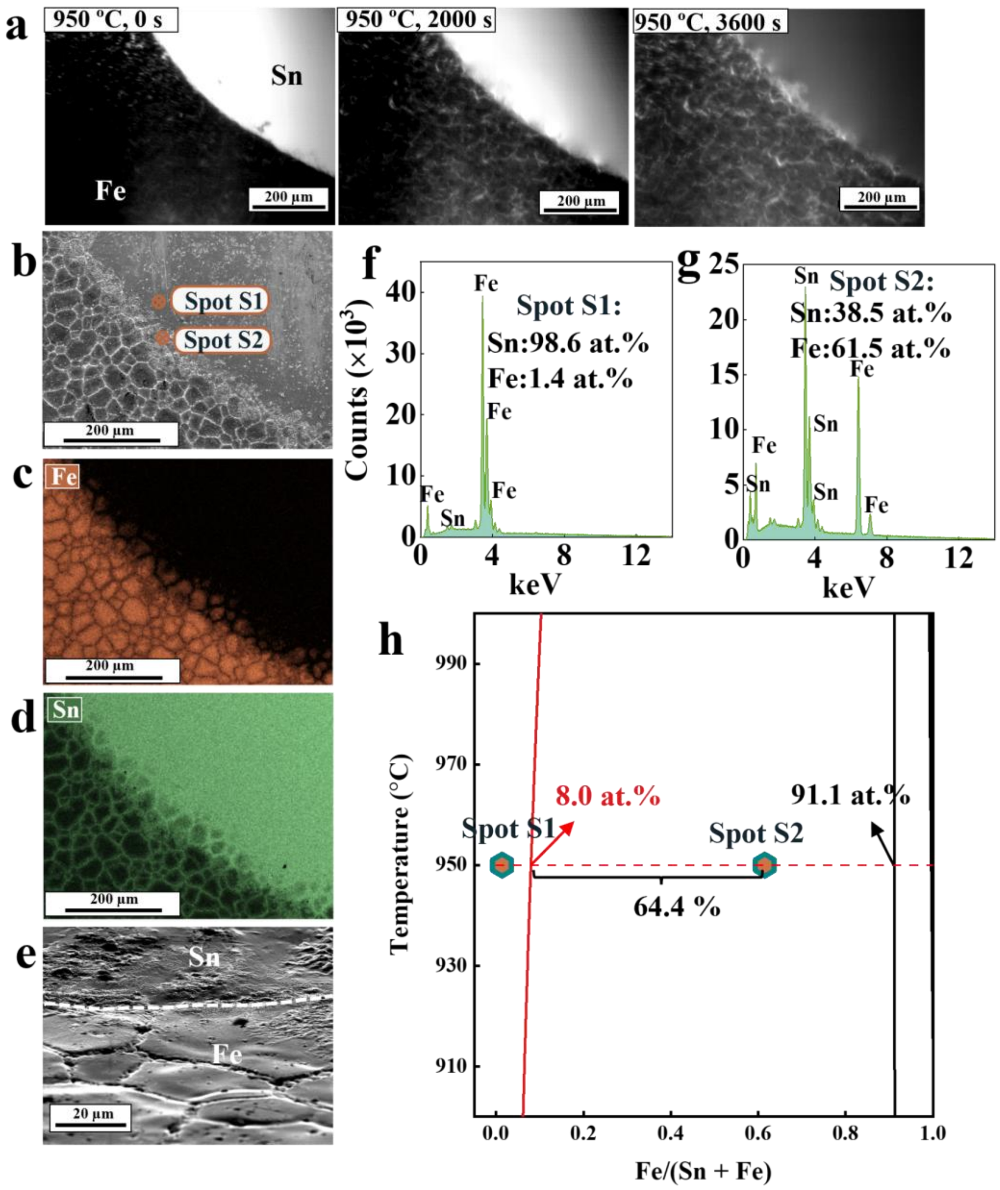

Supplementary Fig. 14 | Spreading behavior and quenched microstructures of the Sn/Fe system. a In-situ observations indicates that the contact line (CL) remains stationary during the isothermal stage. **b** Top-view secondary electron (SE) images of the quenched sample reveal the absence of sharp edges at the CL. Energy-dispersive spectroscopy (EDS) mapping shows the minor distribution of (**c**) Fe within (**d**) the Sn droplet. **e** A side-view SE image further confirms the absence of a sharp edge at the CL (white dash line). **f, g** Fe compositions measured near the CL, as marked in (**b**). The corresponding (**h**) phase diagram suggests that the formation of solid solutions at the liquid/solid interface is impeded due to the large solidification composition range. Source data for (**f, g**) are provided as a Source data file.

Ag on Ti at 1150 °C:

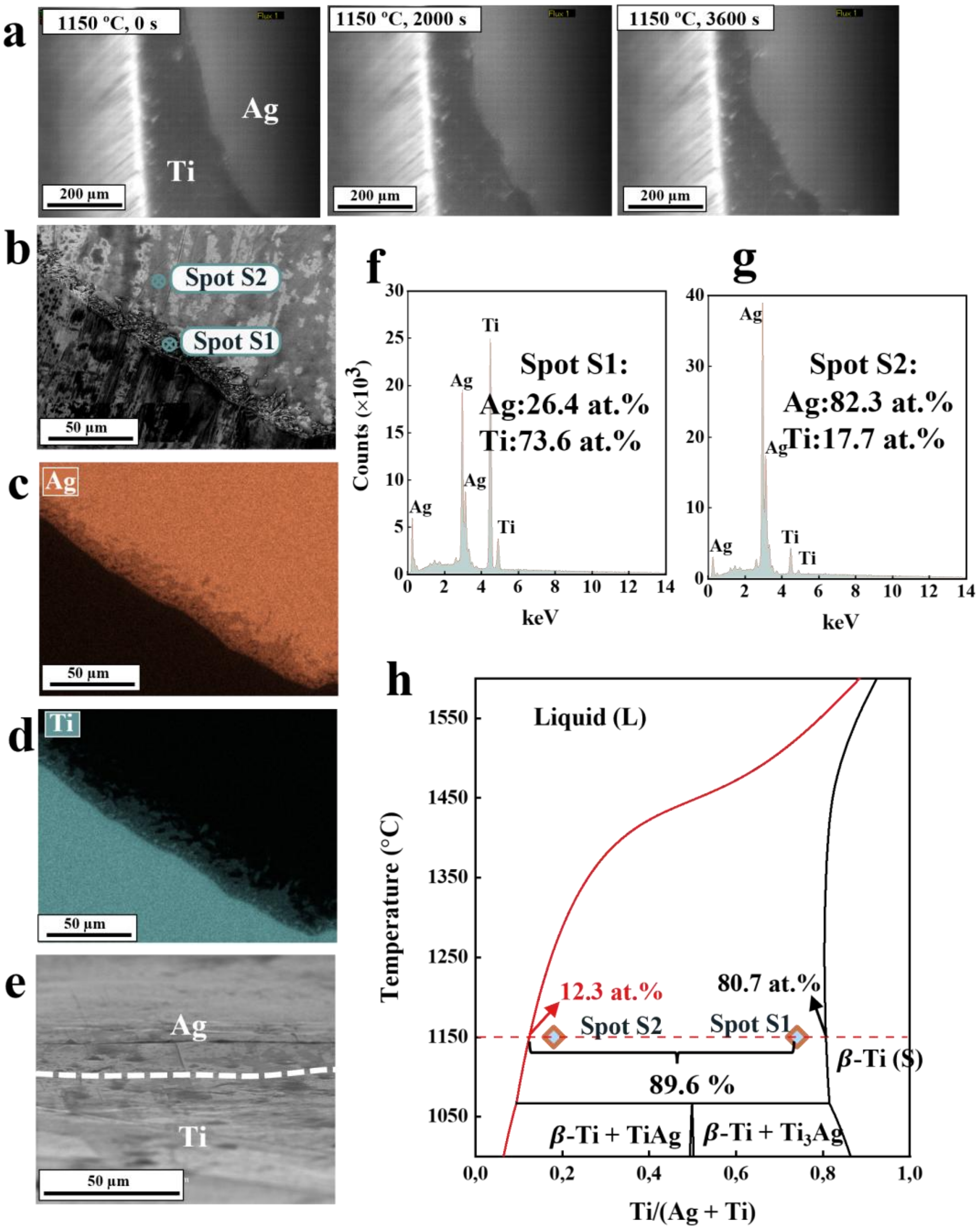

Supplementary Fig. 15 | Spreading behavior and quenched microstructures of the Ag/Ti system. a In-situ observations indicates that the contact line (CL) remains stationary during the isothermal stage. **b** Top-view backscattered electron (SE) images of the quenched sample reveal the absence of sharp edges at the CL. Energy-dispersive spectroscopy (EDS) mapping shows the minor distribution of (**c**) Ti within (**d**) the Ag droplet. **e** A side-view SE image further confirms the absence of a sharp edge at the CL (white dash line). **f, g** Ti compositions measured near the CL, as marked in (**b**). The corresponding (**h**) phase diagram suggests that the formation of solid solutions at the liquid/solid interface is impeded due to the large solidification composition range. Source data for (**f, g**) are provided as a Source data file.

The non-contact heating sessile drop method

The non-contact heating sessile drop method performed with the Thermal Optical Dynamic Wetting Apparatus (TODWA) during wetting experiments can capture the spreading at the initial stage (1000 images per second (s)), and characterize the spreading behavior of the overall droplet from a side view. However, the samples cannot be quenched to evaluate the dissolution and solidification during the isothermal stage due to the use of a thermal-shock sensitive alumina T-junction. To overcome this difficulty, we performed in-situ observation (top view) by conducting contact heating sessile drop method and the subsequent quenching in the Confocal Laser Scanning Microscope (CSLM) with a cooling rate larger than 500 ºC/min. The top-view observation, especially focusing on the movement of the CL, help complementing our interpretation of the spreading process.

TODWA consists of a specialized set of horizontal and vertical alumina (Al_2O_3) tubes which are joined in T-shape, and installed inside the furnace wall (Supplementary Fig. 16a). Molybdenum disilicide elements surround the T-shape tubes and acts as the heat source. The substrate is placed near the center of the inner horizontal tube. Before heating, the Al_2O_3 tubes are thoroughly rinsed by evacuating the tubes to a pressure of approximately 1.7×10^{-2} mbar using a rotary pump (Leybold Sv100b) and then filled with purified Ar gas. This evacuation and purging process is repeated three times to ensure the removal of any remaining oxygen inside the Al_2O_3 tubes. The Ar is purified by passing through an Al_2O_3 tube with Magnesium (Mg) turnings stored inside at 500 °C. The purification is proceeded by the reaction between the gaseous oxygen inside Ar and Mg to form Magnesium oxide (Reaction (S1)):

$$2\text{Mg (s)} + \text{O}_2\text{(g)} = 2\text{MgO (s)} \quad \text{(S1)}$$

Before starting experiments, the temperature difference inside the Al_2O_3 tubes is calibrated by comparing the measurements from two thermocouples (Supplementary Fig. 16a), one is located at a position above the droplet (TC1), while another is placed at the bottom of the inner horizontal tube (TC2). The small difference between them indicates that the temperatures designed for our liquid droplets are accurate (Supplementary Fig. 16b). The solid Cu material is firstly loaded into an Al_2O_3 nozzle mounted on an injector, and then heated separately with the substrate at a rate of 3 ºC/min. When it reaches the desired temperatures, the liquid Cu is squeezed out by a plunger and comes into contact with the substrate. LED light passes through the window, and shadow images of both the Cu droplet and Ni surface are captured by a high-speed camera (Optronis CP70-1-M/C-1000). Purified Ar is flushed through the Al_2O_3 tubes

during the whole process. The oxygen partial pressure inside the chamber is monitored in-situ by an oxygen sensor (15, Rapidox 2100 Gas Analyser). The isothermal stage lasts 1 hour, the furnace then starts to cool down at a rate of 3 ºC/min.

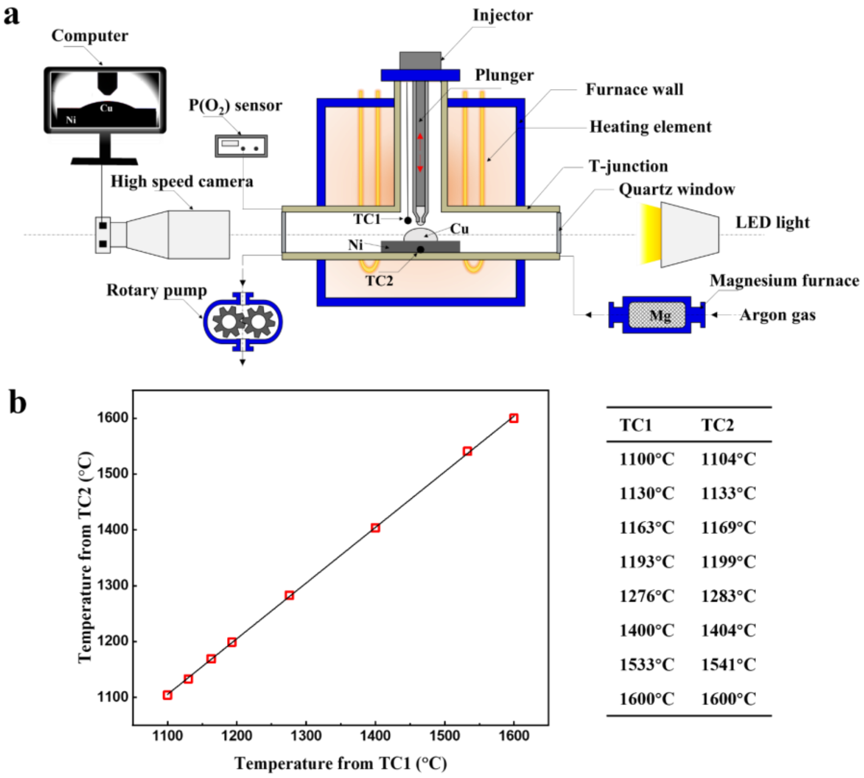

TC1	TC2
1100°C	1104°C
1130°C	1133°C
1163°C	1169°C
1193°C	1199°C
1276°C	1283°C
1400°C	1404°C
1533°C	1541°C
1600°C	1600°C

Supplementary Fig. 16 | Illustration of the non-contact heating experiments. a Schematic of the Thermal Optical Dynamic Wetting Apparatus, TODWA. **b** The minimal temperature difference between thermocouple 1 (TC1) and thermocouple 2 (TC2) in TODWA. The illustration for TODWA in (**a**) was reproduced from Sun et al.[3] with permission.

The Bond number (B_0) balances out the effects of gravity and surface tension, and can be used to characterize droplet size effects (Equation S2).

$$B_0 = \frac{\Delta\rho g R^2}{\sigma_{LV}} \quad \text{(S2)}$$

where $\Delta\rho$ is the difference between the liquid Cu density and that of the surrounded atmosphere. $\Delta\rho$ can be considered to be equal to the density of liquid Cu (7850 kg/m^3 at1150 ºC[4]). g is the gravitational acceleration (9.8 m/s^2). R is the droplet radius of the droplet (Supplementary Fig. 17a). σ_{LV} is the surface tension of liquid Cu taken as 1.3 N/m at 1150 ºC[5].

When $B_0 < 1$, capillary effects dominate over gravity. The droplets tend to adopt a 3D spherical cap shape with a constant curvature (a circle in 2D). The contact angles derived from circle fitting are then valid. In contrast, when $B_0 > 1$, significant droplet flattening occurs due

to gravity. Supplementary Fig. 17a and b show the droplet radius and B_0 for the two Cu/Ni samples at 1150 °C during non-contact heating experiments. Ensuring a consistent droplet size is challenging due to the small volume and mass of droplets (Supplementary Fig. 17c). $B_0 < 1$ is found for both cases, confirming the minimal effects of droplet size. Small droplets (typically 0.01–0.1 g) are preferred for accurate contact angle measurements, as gravity-induced deformation is minimal. Larger droplets (> 1 g) are required only when measuring surface tension[6]. In our case, the droplet mass is 0.13 g, so size effects can be safely neglected.

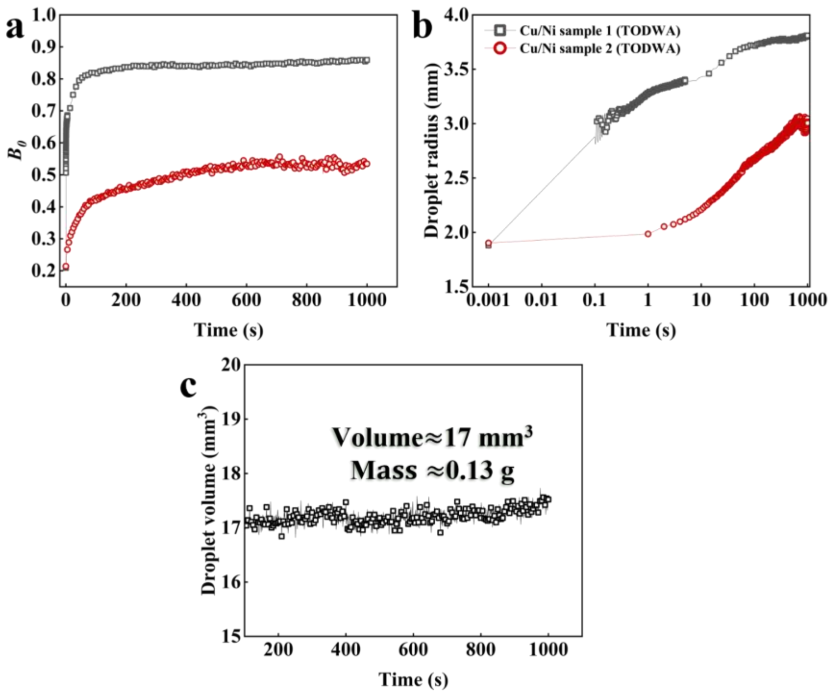

Supplementary Fig. 17 | Size effects of droplets in non-contact heating experiments. a The small Bond number (B_0), calculated from (**b**) the droplet radius, and (**c**) the small droplet volume together confirm that size effects are negligible in the Cu/Ni system. Source data for (**a-c**) are provided as a Source data file.

The spreading process of liquid droplets on solid substrates during the isothermal stage is shown in Figure S2. The droplet is first squeezed out from the nozzle, and then contacts the substrate at the initial moment of the spreading process with an initial contact angle θ_0 (Supplementary Fig. S18a). The droplet then detached from the nozzle and spread on substrates. The relaxation of the dynamic contact angle (θ_d), droplet volume (V_0) and surface area (S_0), as well as the base diameter of the droplets versus time are extracted from the collected pictures (1000 pictures per second with a pixel size of 32 μm) (Supplementary Fig. S18b). After the desired time, the droplets reach a quasi-equilibrium state with contact angles changing less than 0.5 °/min. The contact angle at this time is referred as the quasi-equilibrium contact angle (θ_e), as shown in Supplementary Fig. S18c. The shape evolution of the droplets on top of the

substrates during spreading is analyzed later by using a circle fitting method implemented in the software SCA20[7,8]. As indicated in Supplementary Fig. 18d, the shadow image of droplets is fitted by a circle, and the contact angles, base diameters and the fitting errors between the droplet profile and the circle model are obtained

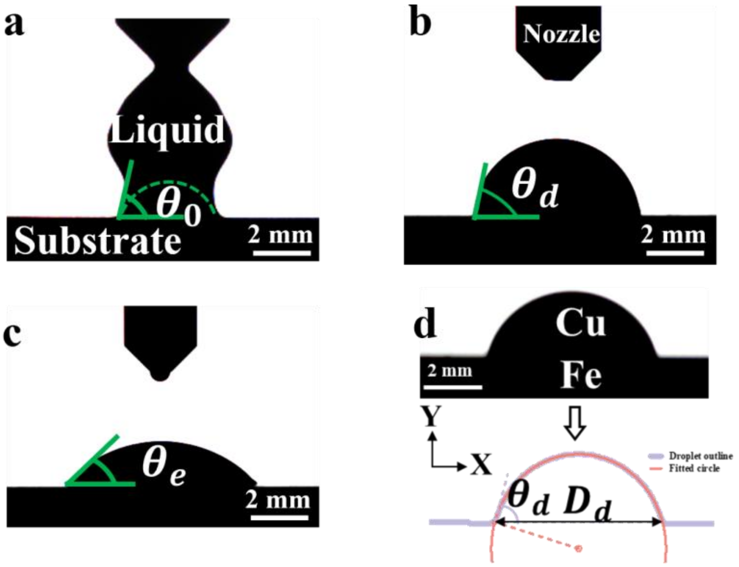

Supplementary Fig. 18 | Illustration of the spreading process. a The liquid droplet contacts the solid substrate at the initial moment of spreading with an initial contact angle θ_0. **b** The dynamic contact angle θ_d during spreading. **c** The equilibrium contact angle θ_e at the equilibrium state. **d** The circle fitting process to obtain contact angles, base diameters and fitting errors between droplet profile and the fitting circle.

The convergence test for atomistic simulations

Convergence tests were conducted to validate the reliability of calculated heats of solution (Figure S17a and b). It can be seen that atom energy remains stable when the cut-off energy exceeds 400 eV (K-point: 7×7×7) and the K-point is beyond 4×4×4 (cut-off energy: 450 eV).

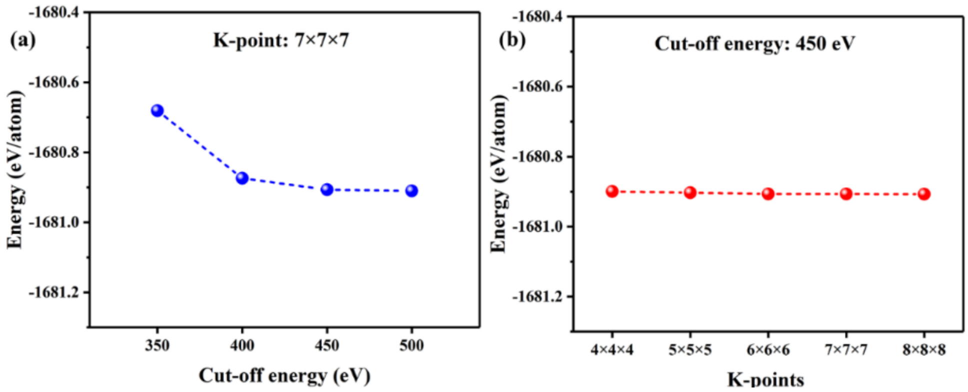

Supplementary Fig. 19 | The convergence test. **a** The cut-off energy, and (**b**) k-points. Source data for (**a, b**) are provided as a Source data file.

Reference

1. Alexandrov, D. V., Galenko, P. K. & Makoveeva, E. V. Solid–liquid interface stability in solidification of a binary mixture under conductive transport and convective flow. *Journal of Applied Physics* **137**, 125110 (2025).
2. Kurz, W., Fisher, D. & Rappaz, M. Fundamentals of solidification. (2023).
3. Sun, Y. *et al.* Effects of oxidation and reactions on reactive wetting behavior in liquid al/solid ni system. *Surfaces and Interfaces* **60**, 106026 (2025).
4. Brillo, J. & Egry, I. Density determination of liquid copper, nickel, and their alloys. *International Journal of Thermophysics* **24**, 1155–1170 (2003).
5. Soda, H., McLean, A. & Miller, W. A. Surface tension measurements of liquid copper droplets in the temperature range 1000 to 1330°C. *Transactions of the Japan Institute of Metals* **18**, 445–454 (1977).
6. Eustathopoulos, N., Nicholas, M. G. & Drevet, B. *Wettability at High Temperatures*. (Pergamon, Amsterdam ; New York, 1999).
7. Zhang, Y. *et al.* Spreading dynamics of molten polymer drops on glass substrates. *Langmuir* **33**, 8447–8454 (2017).
8. Zhang, Y., Zhang, H., Guo, M., De Coninck, J. & Seveno, D. Reactive spreading dynamics of molten polymer liquids. *Macromolecules* **56**, 1111–1121 (2023).
