# Supplementary material for: Thermodynamic framework for assessing dissolutive wetting behaviors in metallic systems
Source: Nat Commun. 2025 Dec 3;17:313. doi: 10.1038/s41467-025-67008-9 (PMC12789441; doi:10.1038/s41467-025-67008-9)
Supplement: Supplementary file 2 — Description of Additional Supplementary Files [file 41467_2025_67008_MOESM2_ESM.pdf]

Description of Additional Supplementary Files

File Name: Supplementary Movie 1

Description: Step flow behavior of Cu on Ni during isothermal stage at 1150 °C

File Name: Supplementary Movie 2

Description: Step flow behavior of Cu on Ti during isothermal stage at 1150 °C

File Name: Supplementary Movie 3

Description: Step flow behavior of Au on Pt during isothermal stage at 1150 °C

File Name: Supplementary Movie 4

Description: Step flow behavior of Ag on Cu during isothermal stage at 1000 °C

File Name: Supplementary Movie 5

Description: Stationary contact line of Sn on Fe during isothermal stage at 950 °C

File Name: Supplementary Data 1

Description: The atomic coordinates for DFT calculations
